# Characterization of temperature-sensitive alleles of anillin-like Mid1 and polo kinase Plo1 in *Schizosaccharomyces pombe*

**DOI:** 10.17912/micropub.biology.001552

**Published:** 2025-03-13

**Authors:** Joshua S. Park, Lesley A. Turner, Kathleen L. Gould, Alaina H. Willet

**Affiliations:** 1 Cell and Developmental Biology, Vanderbilt University School of Medicine, Nashville, TN, US

## Abstract

The *Schizosaccharomyces pombe* anillin-like Mid1 is important for the correct positioning of the cell division site. A key regulator of Mid1 is the polo kinase Plo1 which is important for several mitotic and cytokinetic events including spindle formation and division site placement. Here, we defined the mutations within a set of temperature-sensitive *mid1* and *plo1* alleles and compared the growth and morphological defects of the strains. This work expands the repertoire of *mid1* and *plo1* mutants for studying cytokinesis and highlights the requirement of the Mid1 C2 domain and the Plo1 kinase domain C-terminal lobe as particularly important for cytokinesis.

**Figure 1. Characterization of plo1 and mid1 mutant alleles.**
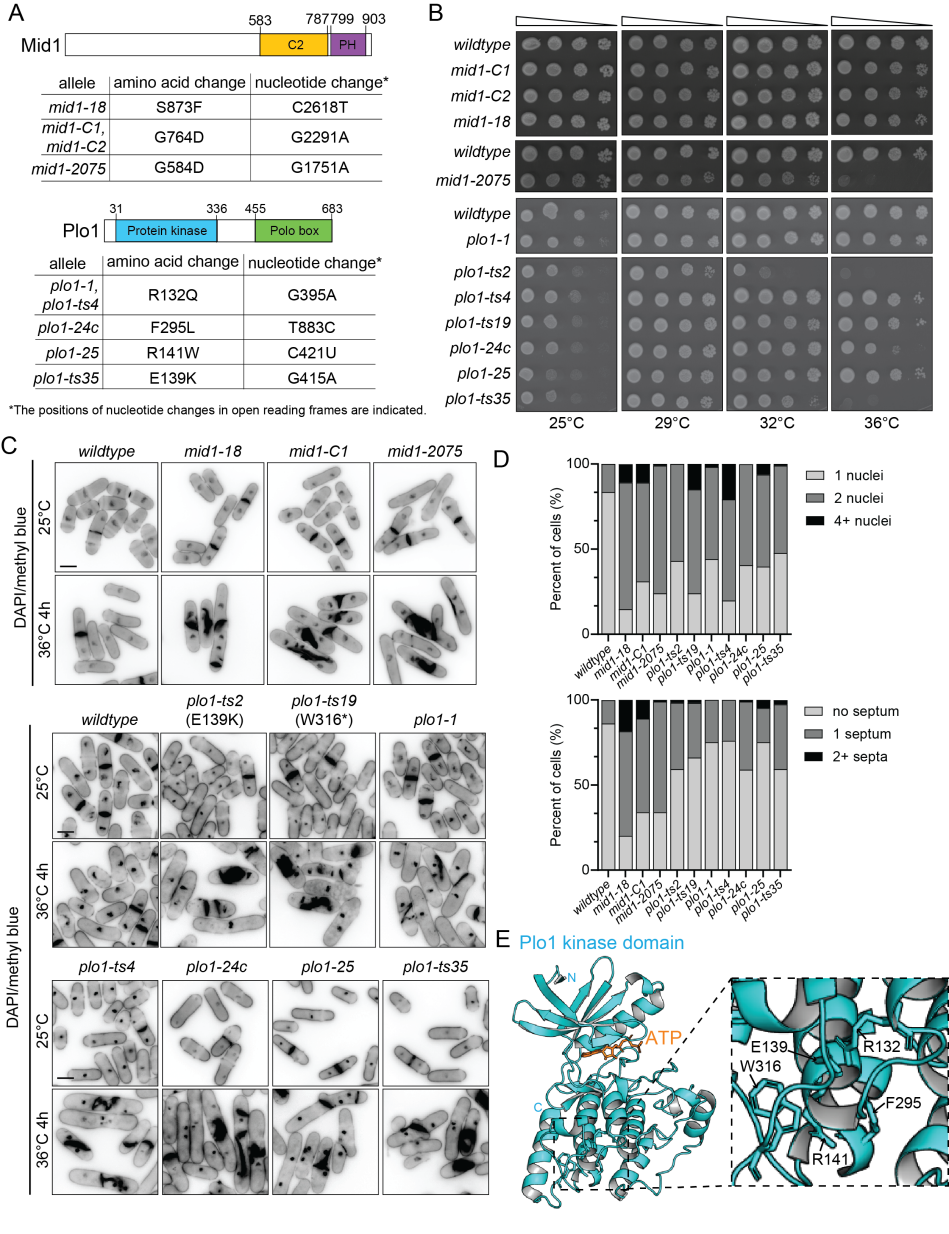
(A) Schematics of Mid1 and Plo1, drawn to scale. The C2 and pleckstrin homology (PH) domains of Mid1 are indicated in yellow and purple, respectively. The protein kinase catalytic domain and polo box domain of Plo1 are indicated in blue and green, respectively. The amino acid and nucleotide substitutions in the indicated temperature-sensitive alleles are provided in the charts. (B) The indicated strains were grown in liquid YE media at 25°C until they reached mid-log phase and then adjusted to the same cell concentration measured by optical density (Moreno et al., 1991). Next, 10-fold serial dilutions were made and 2.5 µL of each was spotted on YE agar plates and incubated at the indicated temperatures for 2-3 days prior to imaging. (C) The indicated strains were grown in liquid YE media. Samples were collected after cells were grown at 25˚C and again after growing the cells for an additional 4 hours at 36˚C. The cells were then fixed and stained with DAPI and methyl blue. Scale bars, 5 µm. (D) The number of nuclei (top) or septa (bottom) per cell was quantified from the same experiment as in C. n ≥ 100 for each. (E) Ribbon diagram of a structural model of *S. pombe* Plo1 kinase domain bound to an ATP molecule using AlphaFold3 (Abramson et al., 2024). The positions of the N- and C-termini of the kinase domain are labelled. A section of the model containing the kinase domain C-terminal lobe is enlarged below and the positions of the mutated residues are indicated.

## Description

The fission yeast *Schizosaccharomyces pombe* utilizes an actin- and myosin-based cytokinetic ring (CR) to accomplish cytokinesis (Cheffings et al., 2016; Glotzer, 2017; Mangione & Gould, 2019). The *S. pombe* CR is built from medial cytokinetic nodes established by the anillin-like Mid1 protein (reviewed in Rincon & Paoletti, 2012). These nodes condense into a coherent ring structure that will eventually constrict concomitant with septum ingression (reviewed in Willet et al., 2015). The precise spatial and temporal ordering of mitotic and cytokinetic events is critical for the faithful segregation of the genetic material into daughter cells. One mechanism by which mitosis and cytokinesis are coordinated is through the action of mitotic kinases that phosphorylate substrates in a cell cycle-dependent manner. One such enzyme is the *S. pombe* polo kinase Plo1 which operates downstream of Cdk1 to promote successful mitosis and cytokinesis (Ohkura et al., 1995; Tanaka et al., 2001). One of Plo1 's substrates is Mid1 and Plo1-dependent phosphorylation of Mid1 promotes the nuclear export of Mid1 (Almonacid et al., 2011; Bähler et al., 1998; Ohkura et al., 1995). This ensures that Mid1 can localize to the medial cytokinetic nodes at mitotic entry to promote CR formation (Almonacid et al., 2009, 2011). Here, we examine *mid1* and *plo1* temperature-sensitive alleles obtained from various genetic screens, some previously uncharacterized and many lacking information as to the sites of mutations (Bähler et al., 1998; Balasubramanian et al., 1998; MacIver et al., 2003).

To better understand the nature of the *mid1* and *plo1* alleles, the respective open reading frames were amplified from nine strains ( *mid1-18, mid1-C1,* *mid1-C2, mid1-2075, plo1-1, plo1-ts4, plo1-24c, plo1-25* and *plo1-ts35* ) in which the relevant mutations have not been reported (Rutherford et al., 2024) and the PCR products sequenced. In each case, a single point mutation was detected ( Figure 1A ). *mid1-18* encodes a serine to phenylalanine substitution at position 873 (S873F), both *mid1-C1* and *mid1-C2* encode a glycine to aspartic acid substitution at position 764 (G764D) and *mid1-2075* encodes a glycine to aspartic acid substitution at position 584 ( Figure 1A ). All of the mutated amino acids reside in structured regions of the protein ( Figure 1A ). Another previously characterized *mid1* allele, *mid1-366* , encodes a different mutation within the C2 domain (G718D) (Chang et al., 1996; Sun et al., 2015). Thus, our results bring the total number of distinct *mid1* temperature-sensitive alleles to four.

Two *plo1* alleles ( *plo1-1* and *plo1-ts4)* encode the same mutation causing an arginine to glutamine change at residue 132 (R132Q). *plo1-24c* encodes a mutation resulting in a change at residue 295 from phenylalanine to leucine, *plo1-25* encodes an arginine to tryptophan substitution at residue 141 (R141W) and *plo1-ts35* encodes a glutamic acid to lysine change at position 139 (E139K) ( Figure 1A ). E139K is the same mutation previously identified in the *plo1-ts2* allele (MacIver et al., 2003). Thus, in addition to two previously sequenced temperature-sensitive *plo1* alleles ( *plo1-ts2* and *plo1-ts19* ), there are three additional distinct temperature-sensitive *plo1* alleles.

To examine the *mid1* and *plo1* mutant cohorts further, we compared the temperature-sensitivity of each strain with a growth assay. *mid1-18, mid1-C1 and mid1-C2* grew similar to wildtype at all temperatures but *mid1-2075* showed almost no growth at 36°C ( Figure 1B ). Two *plo1* alleles, *plo1-ts2* and *plo1-ts35,* had almost no growth at 36°C and reduced growth at 32°C, and all other *plo1* alleles were slightly impaired in growth compared to wildtype at 36°C ( Figure 1B ). These results are consistent with previously published growth assays in which some of the mutants were examined (MacIver et al., 2003; Petersen & Hagan, 2005; Rachfall et al., 2014).

Finally, to visualize and compare the cell phenotypes, we examined each mutant by staining for nuclei and septa after the cells were grown at 25°C and then shifted to 36°C for 4 hours. We found that all temperature sensitive alleles looked similar to wildtype at 25°C, but at 36°C the cells were multinucleated with abnormal septa present ( Figure 1C- D). Our data are consistent with previous descriptions of the subset of previously studied alleles (Bähler et al., 1998; Balasubramanian et al., 1998; Bhutta et al., 2014; Huang et al., 2008; MacIver et al., 2003; Wachtler et al., 2006).

All *mid1* temperature-sensitive alleles displayed phenotypes similar to *mid1∆* (Chang et al., 1996; Sohrmann et al., 1996), suggesting that they are loss-of-function alleles. *mid1-18* has a mutation in the PH domain while *mid1-C1* , *mid1-C2, mid1-2075* and *mid1-366* contain mutations in the C2 domain ( Figure 1A ). The C2 domain appears to directly bind the plasma membrane and is required for Mid1 function (Lee & Wu, 2012; Sun et al., 2015). Cells with a mutant Mid1 lacking the entire PH domain have only mild cell division site positioning defects, thus the function of the PH domain is not fully clear (Lee & Wu, 2012; Paoletti & Chang, 2000). It has been suggested that the PH domain may be important for protein stability and/or play a role in membrane binding in collaboration with the C2 domain, like human anillin (Hall et al., 2024; Liu et al., 2012; Sun et al., 2015). Taken together, we speculate that the *mid1-18* allele produces a less stable protein at high temperatures while the other alleles disrupt C2 domain function.

Plo1 has two essential domains, the N-terminal kinase domain and the C-terminal polo box domain (PBD) (Reynolds & Ohkura, 2003) ( Figure 1A ). While the kinase domain phosphorylates protein substrates, the PBD binds both substrates and protein partners to direct subcellular localization (Park et al., 2010). Interestingly, all mutations encoded by the *plo1* temperature sensitive alleles map within the kinase domain and none are within the PBD ( Figure 1A ). The one unique allele previously sequenced is *plo1-ts19* which contains an early stop codon at tryptophan 316 (MacIver et al., 2003). The W316* mutation truncates an ɑ-helix within the kinase domain and eliminates an unstructured region and the PBD (MacIver et al., 2003) which is a surprising result given the reported requirement for the PBD for Plo1 function (Reynolds & Ohkura, 2003).

To follow up these observations, we used AlphaFold3 to model the Plo1 kinase domain with an ATP molecule (Abramson et al., 2024), and mapped the mutated residues encoded by the *plo1* alleles onto the predicted structure. We found that all of the mutated residues clustered in the C-terminal lobe of the kinase domain ( Figure 1E ). Deciphering if ATP binding, substrate binding or other protein partner binding is defective in these *plo1* alleles will be exciting direction for future studies. Further, how cells tolerate a lack of the Plo1 PBD will be an interesting avenue of study.

## Methods

Yeast methods

*S. pombe* strains were grown in yeast extract (YE) and standard *S. pombe* mating, sporulation, and tetrad dissection techniques were used to construct new strains (Moreno et al., 1991).

Molecular biology methods

The *mid1* open reading frames from *mid1-C1* and *mid1-C18* cells were amplified by generating a PCR product with an oligonucleotide 74 bp upstream of the start site (GTTGTACTTCAGGGTGCTTA) and an oligonucleotide 380 bp downstream of the stop codon (AGGTTCTCCATCTCATGGCT) (Integrated DNA technologies). The *mid1* open reading frame was amplified from *mid1-C2* cells using two sets of overlapping oligonucleotides. The first PCR product was generated with the above-mentioned oligonucleotide 74 bp upstream of the start site and an oligonucleotide 1350 bp into the open reading frame (GTTGCATTGATGGGTGACGT). The second PCR product was produced with an oligonucleotide 1200 bp within the open reading frame (GTATGGTCATGGATCTGTAACG) and the above-mentioned oligonucleotide 380 bp downstream of the stop codon.

The *plo1* alleles were amplified by generating two PCR products. One PCR reaction used an oligonucleotide 40 bp upstream of the start site (GCAACCACTTTGTTTACCCTCA) and an oligonucleotide 1100 bp into the open reading frame of *plo1* (TGGACTTAAAACACTTGGTAATATTCG) (Integrated DNA technologies). The second PCR reaction used an oligonucleotide 900 bp into the open reading frame (TCCAGATGAAATTTTACATTCAATGCCT) with an oligonucleotide 20 bp downstream of the stop codon (GCATAGTAACTTAACGCCCAAGTA).

The PCR products were sequenced by Plasmidsaurus using Oxford Nanopore Technology with custom analysis and annotation.

Microscopy and image analysis

Strains for fixed-cell imaging experiments were grown at 25°C in YE and then shifted to 36°C for 3 hours. Cells were fixed with 70% ethanol for DAPI and methyl blue (MB) staining as described previously (Roberts-Galbraith et al., 2009). Images were acquired using a Zeiss Axio Observer inverted epifluorescence microscope with Zeiss 63× oil (1.46 NA) objective and captured using Zeiss ZEN 3.0 (Blue edition) software. A singular medial Z slice was obtained. All images were further processed using ImageJ (Schindelin et al., 2012).

AlphaFold3 structural prediction

Protein structure predictions were generated with the AlphaFold3 server (Abramson et al., 2024) and visualized using the PyMOL molecular graphics system (version 3.0, Schrodinger, LLC).

## Reagents

The strains used in this study and their genotypes are listed below.

**Table:** 

**Strain**	**Genotype**	**Source**
KGY246	*ade6-M210 leu1-32 ura4-D18* *h ^-^*	Lab stock
KGY1001	* plo1-1 ura4-D18 leu1-32 ade6-M21X h ^+^*	*Bahler et al., 1998*
KGY846	* plo1-ts4:ura4 ^+^ ura4-D18 leu1-32 ade6-M210 h ^+^*	*MacIver et al., 2003*
KGY1460	* plo1-24C leu1-32 ura4-D18 ade6-M216 his3-D1 h ^+^*	*Bahler et al., 1998*
KGY16150-2	* plo1-ts35:ura4 ^+^ ura4-D18 leu1-32 ade6-M210 h- *	*Anderson et al., 2002*
KGY15085	* plo1-25 ade6-M21X leu1-32 ura4-D18 h ^+^*	*Bahler et al., 1998*
KGY16148-2	* plo1-ts2:ura4 ^+^ ura4-D18 leu1-32 ade6-M210 h ^-^*	*MacIver et al., 2003*
KGY16149-2	* plo1-ts19:ura4 ^+^ ura4-D18 leu1-32 ade6-M210 h ^-^*	*MacIver et al., 2003*
KGY1058	* mid1-C1 ura1 leu1-32 mam2::LEU2 ade6-216 h ^90^*	Balasubramanian et al., 1998
KGY1059	*mid1-C2 ura1 leu1-32 mam2::LEU2 ade6-216*	Balasubramanian et al., 1998
KGY19270	* mid1-18 ura4-D18 leu1-32 ade6-21X h ^-^*	Balasubramanian et al., 1998
KGY442-2	* mid1-C1 ura4-D18 h ^90^*	This study
KGY436-2	* mid1-C2 ura4-D18 h ^-^*	This study
KGY3717	* mid1-2075 ura4-D18 ade6-21X h ^-^*	This study

## References

[R1] Abramson Josh, Adler Jonas, Dunger Jack, Evans Richard, Green Tim, Pritzel Alexander, Ronneberger Olaf, Willmore Lindsay, Ballard Andrew J., Bambrick Joshua, Bodenstein Sebastian W., Evans David A., Hung Chia-Chun, O’Neill Michael, Reiman David, Tunyasuvunakool Kathryn, Wu Zachary, Žemgulytė Akvilė, Arvaniti Eirini, Beattie Charles, Bertolli Ottavia, Bridgland Alex, Cherepanov Alexey, Congreve Miles, Cowen-Rivers Alexander I., Cowie Andrew, Figurnov Michael, Fuchs Fabian B., Gladman Hannah, Jain Rishub, Khan Yousuf A., Low Caroline M. R., Perlin Kuba, Potapenko Anna, Savy Pascal, Singh Sukhdeep, Stecula Adrian, Thillaisundaram Ashok, Tong Catherine, Yakneen Sergei, Zhong Ellen D., Zielinski Michal, Žídek Augustin, Bapst Victor, Kohli Pushmeet, Jaderberg Max, Hassabis Demis, Jumper John M. (2024). Accurate structure prediction of biomolecular interactions with AlphaFold 3. Nature.

[R2] Almonacid Maria, Celton-Morizur Séverine, Jakubowski Jennifer L., Dingli Florent, Loew Damarys, Mayeux Adeline, Chen Jun-Song, Gould Kathleen L., Clifford Dawn M., Paoletti Anne (2011). Temporal Control of Contractile Ring Assembly by Plo1 Regulation of Myosin II Recruitment by Mid1/Anillin. Current Biology.

[R3] Almonacid Maria, Moseley James B., Janvore Julie, Mayeux Adeline, Fraisier Vincent, Nurse Paul, Paoletti Anne (2009). Spatial Control of Cytokinesis by Cdr2 Kinase and Mid1/Anillin Nuclear Export. Current Biology.

[R4] Bähler Jürg, Steever Alexander B., Wheatley Sally, Wang Yu-li, Pringle John R., Gould Kathleen L., McCollum Dannel (1998). Role of Polo Kinase and Mid1p in Determining the Site of Cell Division in Fission Yeast. The Journal of Cell Biology.

[R5] Balasubramanian Mohan K, McCollum Dannel, Chang Louise, Wong Kelvin C Y, Naqvi Naweed I, He Xiangwei, Sazer Shelley, Gould Kathleen L (1998). Isolation and Characterization of New Fission Yeast Cytokinesis Mutants. Genetics.

[R6] Bhutta Musab, McInerny Christopher, Gould Gwyn (2014). ESCRT Function in Cytokinesis: Location, Dynamics and Regulation by Mitotic Kinases. International Journal of Molecular Sciences.

[R7] Chang Fred, Woollard Alison, Nurse Paul (1996). Isolation and characterization of fission yeast mutants defective in the assembly and placement of the contractile actin ring. Journal of Cell Science.

[R8] Cheffings Thomas H., Burroughs Nigel J., Balasubramanian Mohan K. (2016). Actomyosin Ring Formation and Tension Generation in Eukaryotic Cytokinesis. Current Biology.

[R9] Glotzer Michael (2016). Cytokinesis in Metazoa and Fungi. Cold Spring Harbor Perspectives in Biology.

[R10] Hall Aaron R., Choi Yeol Kyo, Im Wonpil, Vavylonis Dimitrios (2024). Anillin-related Mid1 as an adaptive and multimodal contractile ring anchoring protein: A simulation study. Structure.

[R11] Huang Yinyi, Yan Hongyan, Balasubramanian Mohan K. (2008). Assembly of normal actomyosin rings in the absence of Mid1p and cortical nodes in fission yeast. The Journal of Cell Biology.

[R12] Lee I-Ju, Wu Jian-Qiu (2012). Characterization of Mid1 domains for targeting and scaffolding in fission yeast cytokinesis. Journal of Cell Science.

[R13] Liu Jinghe, Fairn Gregory D., Ceccarelli Derek F., Sicheri Frank, Wilde Andrew (2012). Cleavage Furrow Organization Requires PIP2-Mediated Recruitment of Anillin. Current Biology.

[R14] MacIver Fiona H., Tanaka Kayoko, Robertson Alasdair M., Hagan Iain M. (2003). Physical and functional interactions between polo kinase and the spindle pole component Cut12 regulate mitotic commitment in
*S. pombe*. Genes & Development.

[R15] Mangione M. C., Gould Kathleen L. (2019). Molecular form and function of the cytokinetic ring. Journal of Cell Science.

[R16] Moreno Sergio, Klar Amar, Nurse Paul (1991). [56] Molecular genetic analysis of fission yeast Schizosaccharomyces pombe. Guide to Yeast Genetics and Molecular Biology.

[R17] Ohkura H, Hagan I M, Glover D M (1995). The conserved Schizosaccharomyces pombe kinase plo1, required to form a bipolar spindle, the actin ring, and septum, can drive septum formation in G1 and G2 cells.. Genes & Development.

[R18] Paoletti Anne, Chang Fred (2000). Analysis of mid1p, a Protein Required for Placement of the Cell Division Site, Reveals a Link between the Nucleus and the Cell Surface in Fission Yeast. Molecular Biology of the Cell.

[R19] Park Jung-Eun, Soung Nak-Kyun, Johmura Yoshikazu, Kang Young H., Liao Chenzhong, Lee Kyung H., Park Chi Hoon, Nicklaus Marc C., Lee Kyung S. (2010). Polo-box domain: a versatile mediator of polo-like kinase function. Cellular and Molecular Life Sciences.

[R20] Petersen Janni, Hagan Iain M. (2005). Polo kinase links the stress pathway to cell cycle control and tip growth in fission yeast. Nature.

[R21] Rachfall Nicole, Johnson Alyssa E., Mehta Sapna, Chen Jun-Song, Gould Kathleen L. (2014). Cdk1 promotes cytokinesis in fission yeast through activation of the septation initiation network. Molecular Biology of the Cell.

[R22] Reynolds Nicola, Ohkura Hiroyuki (2003). Polo boxes form a single functional domain that mediates interactions with multiple proteins in fission yeast polo kinase. Journal of Cell Science.

[R23] Rincon Sergio A., Paoletti Anne (2016). Molecular control of fission yeast cytokinesis. Seminars in Cell & Developmental Biology.

[R24] Roberts-Galbraith Rachel H., Chen Jun-Song, Wang Jianqiu, Gould Kathleen L. (2009). The SH3 domains of two PCH family members cooperate in assembly of the
*Schizosaccharomyces pombe*
contractile ring. The Journal of Cell Biology.

[R25] Rutherford Kim M, Lera-Ramírez Manuel, Wood Valerie (2024). PomBase: a Global Core Biodata Resource—growth, collaboration, and sustainability. GENETICS.

[R26] Schindelin Johannes, Arganda-Carreras Ignacio, Frise Erwin, Kaynig Verena, Longair Mark, Pietzsch Tobias, Preibisch Stephan, Rueden Curtis, Saalfeld Stephan, Schmid Benjamin, Tinevez Jean-Yves, White Daniel James, Hartenstein Volker, Eliceiri Kevin, Tomancak Pavel, Cardona Albert (2012). Fiji: an open-source platform for biological-image analysis. Nature Methods.

[R27] Sohrmann M, Fankhauser C, Brodbeck C, Simanis V. 1996. The dmf1/mid1 gene is essential for correct positioning of the division septum in fission yeast. Genes & development. 10: 2707. 10.1101/gad.10.21.2707 8946912

[R28] Sun Lingfei, Guan Ruifang, Lee I-Ju, Liu Yajun, Chen Mengran, Wang Jiawei, Wu Jian-Qiu, Chen Zhucheng (2015). Mechanistic Insights into the Anchorage of the Contractile Ring by Anillin and Mid1. Developmental Cell.

[R29] Tanaka Kayoko, Petersen Janni, MacIver Fiona, Mulvihill Daniel P., Glover David M., Hagan Iain M. (2001). The role of Plo1 kinase in mitotic commitment and septation in Schizosaccharomyces pombe. The EMBO Journal.

[R30] Wachtler Volker, Huang Yinyi, Karagiannis Jim, Balasubramanian Mohan K. (2006). Cell Cycle-dependent Roles for the FCH-Domain Protein Cdc15p in Formation of the Actomyosin Ring in
*Schizosaccharomyces pombe*. Molecular Biology of the Cell.

[R31] Willet Alaina H, McDonald Nathan A, Gould Kathleen L (2015). Regulation of contractile ring formation and septation in Schizosaccharomyces pombe. Current Opinion in Microbiology.

